# Establishment of a reliable in-vivo model of implant-associated infection to investigate innovative treatment options

**DOI:** 10.1038/s41598-022-07673-8

**Published:** 2022-03-10

**Authors:** C. Kreis, F. K. Aschenbrenner, D. Günther, N. Tholema-Hans, J. Koeppe, S. B. Rosslenbroich, M. J. Raschke, T. Fuchs

**Affiliations:** 1 Department of Trauma, Hand and Reconstructive Surgery, University Hospital of Muenster, Waldeyerstrasse 1, 48149 Muenster, Germany; 2 Department of Anesthesia, Hospital Lippe Detmold, Detmold, Germany; 3 Department of Orthopaedic Surgery, Trauma Surgery and Sports Medicine, Cologne Merheim Medical Center, Witten/Herdecke University, Cologne, Germany; 4 Institute of Biostatistics and Clinical Research, University of Muenster, Muenster, Germany; 5 Department of Trauma and Reconstructive Surgery, Vivantes Clinic Friedrichshain, Berlin, Germany

**Keywords:** Health care, Medical research

## Abstract

The increasing number of implant-associated infections and of multiresistant pathogens is a major problem in the daily routine. In the field of osteomyelitis, it is difficult to manage a valid clinical study because of multiple influencing factors. Therefore, models of osteomyelitis with a simulation of the pathophysiology to evaluate treatment options for implant-associated infections are necessary. The aim of this study is to develop a standardized and reproducible osteomyelitis model in-vivo to improve treatment options. This study analyses the influence of a post-infectious implant exchange one week after infection and the infection progress afterward in combination with a systemic versus a local antibiotic treatment in-vivo. Therefore, the implant exchange, the exchange to a local drug-delivery system with gentamicin, and the implant removal are examined. Furthermore, the influence of an additional systemic antibiotic therapy is evaluated. An in-vivo model concerning the implant exchange is established that analyzes clinic, radiologic, microbiologic, histologic, and immunohistochemical diagnostics to obtain detailed evaluation and clinical reproducibility. Our study shows a clear advantage of the combined local and systemic antibiotic treatment in contrast to the implant removal and to a non-combined antibiotic therapy. Group genta/syst. showed the lowest infection rate with a percentage of 62.5% concerning microbiologic analysis, which is in accordance with the immunohistochemical, cytochemical, histologic, and radiologic analysis. Our in-vivo rat model has shown valid and reproducible results, which will lead to further investigations regarding treatment options and influencing factors concerning the therapy of osteomyelitis and implant-associated infections.

## Introduction

Osteomyelitis is an inflammatory process caused by infective microorganisms that can develop towards an acute or chronic process and is accompanied by bone destruction^1^. The increasing number of implant-associated infections in combination with an increasing number of multiresistant pathogens resembles a major problem for the treating physician^2,3^. The prevalence of hematogenous and recurrent osteomyelitis decreases with increasing age, whereas the incidence of exogen bone infections, e.g., after joint replacement, complicated surgical intervention, and wound infections, increases^4,5^. Up to 33% of the patients develop an implant-associated infection after suffering an open fracture, as those fractures are bacteria-contaminated with a rate of 50–70%^6,7^. Implant-associated infections are often caused by *Staphylococcus epidermidis* (*S. epidermidis*) and *Staphylococcus aureus* (*S. aureus*), which is the most common pathogen of osteomyelitis with a percentage of 80%, among other bacteria, e.g., *Streptococcus ssp*. and gram-negative bacteria^8–10^.

The standard treatment for acute bone infection in its very early stages is antibiotic therapy. For the treatment of implant-associated and chronic stages of infection, a combined therapy becomes necessary, which includes a combination of local and systemic antibiotics to reach high local concentrations, surgical debridement, implant removal, fracture stabilization, and soft tissue management. Surgical debridement is known to be the most effective treatment component. The most efficient treatment outcomes in *S. aureus-related* implant-associated infections can be achieved by a combined antibiotic treatment combining rifampicin and another antibiotic agent^1,2,8,9,11^. Nevertheless, the combined treatment path of antibiotic and surgical treatment still fails with a rate of 20%, rates of persistent infection are described with a rate up to 33%, and reinfection rates of 40%^6^.

Reasons for this high failure rate are the properties of *S. aureus*. With the help of a variety of virulence factors, this pathogen has the ability to invade host cells, including osteoblasts, and to persist intracellularly, where antibiotic activity decreases—even of substances that are known to accumulate intracellularly^1–3,8,9,12^. *S. aureus* also has the ability to change its phenotype into a small colony variant (SCV), which demonstrates a subpopulation with low metabolism and the ability for long-term persistence intracellularly. SCVs also show an increased resistance against antibiotics, especially against aminoglycosides and trimethoprime/sulfomethaxzol. Some studies even postulate an SCV-induction by subinhibitory doses of aminoglycosides and trimethoprime/sulfomethaxzol^2,3,8,13–16^. Bacteria that cause implant-associated infections are also able to adhere to foreign material and to form a biofilm matrix on its surfaces, which consists of polysaccharides and proteins and serves as a diffusion barrier that inhibits the diagnose and eradication of the pathogens, that prevents the phagocytosis and weakens the function of B- and T-lymphocytes. Furthermore, bacteria that are included in a biofilm show a reduced metabolism. The biofilm itself acts as a nutrition medium and promotes cell communication as well as the exchange of virulence factors^1–3,8,17–21^.

Antibiotic-loaded bone cement is one effective option to achieve high antibiotic levels locally as it works as a drug-delivery system^22–24^. Polymethylmetacrylate (PMMA)-bone cement loaded with antibiotic substances—in most cases gentamicin or vancomycin—is an established treatment for infected endoprostheses and implant-associated infections^22–26^. Besides a local delivery system at the site of infection with limited blood flow, antibiotic-loaded bone cement serves for dead space management. Nevertheless, the PMMA-spacer has to be removed and may lead to negative side effects like bacterial resistance and recurrence of infection. Overall, 70–90% of the cases proved a successful treatment^6^.

The use of coated implants as another option of a drug-delivery-system has the opportunity to prevent biofilm-synthesis and the development of implant-associated infections, whereas a poly-d, l-lactid (PDLLA) coating serves as a carrier and biodegradable material limits the antibiotic release so that an initial peak can be achieved 6 h after implantation followed by a continuous antibiotic release^27–30^. In contrast to antibiotic-loaded bone cement, no removal is necessary. Furthermore, implant coating increases the mechanical stability of the implant and makes it much more difficult for bacteria to adhere to the material. Studies and clinical experience with the use of coated implants reveal promising results to reduce and prevent implant-associated infections by this method^31,32^. Different in-vivo models have already evaluated this method^27,28^.

Our working group focuses on local antibiotic treatment in cases of implant-associated infections. Therefore, we developed this in-vivo study to analyze the influence of a post-infectious implant exchange one week after infection and the infection progress afterward. Furthermore, the influence of the exchange to a local drug-delivery system with gentamicin and the implant removal are examined as well as the influence of an additional systemic antibiotic therapy. We focused on *S. aureus* still being the major pathogens that cause implant-associated infections^9,10^.

The study analyses the clinical, radiological, microbiological, histological, and immunohistochemical outcomes to obtain detailed evaluation and clinical reproducibility. Our findings provide the opportunity to investigate further treatment options, potent treatment alternatives, and influencing factors concerning the therapy of implant-associated infections.

## Material and methods

### Bacterial strains and preparation of bacterial inocula

Overnight cultures of *Staphylococcus aureus* strain Rosenbach ATCC 49230 were prepared and incubated in 5 ml glucose broth at 37 °C. After various washing steps with phosphate-buffered saline (PBS) and centrifugation at 4000 rpm, the concentration was determined by OD (optical density) measurement. The inoculum was resuspended in PBS to get a concentration with an amount of 5 × 10^4^ bacteria/ml. The concentration of the inoculum was confirmed by plating and determination of the CFU/ml.

### In-vivo model

All in-vivo examinations were performed after approval by the district government Muenster (project number: Ra2/109/04; 2-Q002; file number: 50.0835.1.0) and its ethics committee. All methods were carried out in accordance with relevant guidelines and regulations and in accordance with ARRIVE guidelines. Female Sprague–Dawley rats were obtained with an age of 12 weeks and an average bodyweight of 230–300 g. In total, 120 animals were used for this experimental study with a drop-out rate of 11.76%. Rats were anesthetized intraperitoneally with 10% ketamine (80–100 mg per kg body weight) and 2% xylazine (12 mg per kg body weight). After individual marking, each rat was weighed, rectal body temperature was measured, and 1 ml blood was taken. The operation was performed at the left hindlimb of each rat under sterile conditions. After skin incision at the height of the tibial tuberosity medio-proximally the medullary space of the rat's tibia was opened by drilling with titanium k-wires with diameters of 0.8 mm and 1 mm right below the patellar tendon. 20 μl of the prepared *S. aureus* inoculum was applied to the channeled medullary space. Thereupon an uncoated titanium implant with a diameter of 0.8 mm, which was manufactured by DePuy Synthes/Switzerland, was applied to the medullary space of the channeled tibia. The implant was shortened adequately, the skin incision was sutured, and a sterile bandage was applied. Postoperative analgesia was reached by treatment with Rimadyl® per os (0.16 ml/500 ml drinking water) and subcutaneously (4 mg/kg body weight) daily. Seven days after the first operation, a second operation was performed at each animal in the same way concerning anesthesia, implementation, and analgesia. During the second operation (nail exchange = NE), the uncoated implant was removed, the medullary space was rinsed and debrided with 20 ml antiseptic lavasept solution. The animals were then divided into examination groups (Table 1). Concerning the examination group, the debridement was either followed by the implantation of a new, uncoated titanium implant with a diameter of 0.8 mm (**"titan"**) or by the implantation of a coated implant (diameter 0.8 mm) with a combined coating of PDLLA + 10% gentamicine (**"genta"**), which was performed by Synthes/Switzerland, or by leaving the medullary space without an implant (**"expl./syst."**). In order to monitor the position of the implant, the left tibia of each rat was x-rayed (anterior/posterior, lateral) after the first and the second operation. After the second operation, the examination groups were further divided and half of the animals with a new, uncoated titanium implant (**"titan/syst."**), half of the animals with a coated implant (**"genta/syst.”**) and all of the animals without an implant (**"expl./syst.”**) were treated with gentamicine (5 mg/kg bodyweight) systemically for six days by intraperitoneal application once a day. Table 1 gives a detailed overview of the subdivision of all examination groups. Physical examination occurred on day 7, 14, 21, and 28: after sedation with Domitor® (0.15 ml/kg body weight), bodyweight and rectal temperature of each animal was measured, blood was drawn from the right V. tibialis posterior to determine hemoglobin [mg/dl], hematocrit [%] and leukocytes [× 1000/µl] and x-rays of each tibia were performed.

**Table 1:** Subdivision and group size of all examination groups.

Examination group	First operation	Second operation (nail exchange = NE)	Analysis
**"titan "**	Uncoated titanium implant + 20 μl *S. aureus* inoculum *Staphylokokkus aureus*-Suspension (n = 120)	New uncoated titanium implant (n = 24)	Microbiologic analysis (n = 8)
			Immunohistochemical and cytochemical analysis (n = 8)
			Histologic analysis (n = 8)
**"genta "**		Coated implant with a combined coating of PDLLA + 10% gentamicine (n = 24)	Microbiologic analysis (n = 8)
			Immunohistochemical and cytochemical analysis (n = 8)
			Histologic analysis (n = 8)
**“expl./syst.”**		Leaving the medullary space without an implant + gentamicin systemically (n = 24)	Microbiologic analysis (n = 8)
			Immunohistochemical and cytochemical analysis (n = 8)
			Histologic analysis (n = 8)
**“titan/syst.”**		New uncoated titanium implant + gentamicin systemically (n = 24)	Microbiologic analysis (n = 8)
			Immunohistochemical and cytochemical analysis (n = 8)
			Histologic analysis (n = 8)
**“genta/syst.”**		Coated implant with a combined coating of PDLLA + 10% gentamicine + gentamicin systemically (n = 24)	Microbiologic analysis (n = 8)
			Immunohistochemical and cytochemical analysis (n = 8)
			Histologic analysis (n = 8)

### The sacrifice of the animals

All animals were sacrificed on day 28 by an intracardial KCl (10%)-injection after anesthesia with ketamine and xylazine. The tibia of the left hindlimb was removed, the implant was taken out and plated on blood agar. For immunohistochemical analysis, tibiae were placed and fixed with 4% paraformaldehyde solution.

### Microbiologic analysis

The procedure for animals that were randomized for microbiologic analysis differed as 2 ml heart-blood were taken sterile before KCl-injection through a vertical thoracotomy. 1.5 ml were added directly into a blood culture bottle and 0.5 ml were added into an ethylenediamine-tetraacetate (EDTA)-tube. Before the implant was taken out of the tibia, a smear test was performed at the insertion. In order to determine the bacterial load, the implant and also the smear test were plated on blood agar, both then were incubated in glucose bouillon. The tibiae and the kidneys of two animals of each group were homogenized and plated on blood agar in serial dilution. Furthermore, 100 μl of heart blood were also plated on blood agar plates in order to determine bacterial growth. The glucose bouillon was evaluated after 24 h of incubation concerning cloudiness, formation of gas, discoloration, and sediment. In order to identify bacteria as *S. aureus* Pastorex®-tests were performed. Additionally, the number of small colony variants (SCV) is determined and further analyzed by Vitek® 2XL and polymerase chain reaction (PCR) for reasons of identification and resistance. Blood culture bottles were evaluated automatically by BacTec 9250 (BD Diagnostics).

### Immunohistochemical analysis

For immunohistochemical evaluation and cytochemical coloring for the evaluation of interleukines IL-1α, IL-6 and MCP-1 the removed tibiae were fixed in a 4% paraformaldehyde solution for 12-24 h after preparation, decalcification, draining and embedding in paraffin. Samples were sliced axially into layers with a thickness of 5 μm. Decalcification was performed by 20% EDTA-solution combined with 0.2% paraformaldehyde. Draining of the tibiae was reached by washing steps with 0.1 M phosphatebuffer followed by draining with alcohol in different concentrations (70–100%). Coloring of the samples was conducted following the avidin–biotin-comlex-method (ABC-method). In order to unmask antigens, the samples were treated with 1% trypsin. IL-1α and IL-6 coloring was blocked with 10% horse serum whereas MCP-1 coloring was blocked by 10% goat serum to inhibit cross reaction between the secondary antibodies and endogen immunoglobulins. Before applying the secondary biotin and the avidin-alkaline-phosphatase complex the primary antibodies against IL-1α, IL-6 and MCP-1 were put onto the samples diluted in 2% horse/goat serum. After washing with H_2_O slices were counterstained with methyl green. Dehydration was performed by alcohol in ascending concentrations (96–100%). Samples were then added to xylol and were stocked up with a sealing compound (Eukitt®). The specificity of the secondary antibody could be confirmed by negative control staining. Sample components that showed a reaction with the antibody appeared red. Analysis and evaluation of the prepared slices occurred blinded, quantitatively and descriptively with a microscope (BX51, Olympus) with a 10 times enlargement and with the help of Image Pro Plus®-software. Desciptive analysis was done in consideration to the 4 parts of the drill channel (proximal metaphysis, proximal diaphysis, distal diaphysis, distal metaphysis) and in comparison to cytochemical Trap-coloring as well as to the x-ray-pictures of day 28.

### Cytochemical analysis

With the help of cytochemical coloring of the enzyme TRAP (tartrate resistant acid phosphatase), which is located inside the cells of the mononuclear phagocyte's system as biomarker of osteoclasts and inflammatory macrophages^33^, osteoclasts in rats' tibiae were detected. The paraffin slices of the tibiae were colored by a special process with naphtol AS-BI phosphoric acid, acetat solution and tartrate solution mixed with aqua dest. After incubation in the dark at 37 °C fast garnet GBC base solution and sodium nitrite solution were added before counterstaining with hematoxylin solution. Analysis and evaluation occurred analogous to immunohistochemial analysis and also with a 40 times enlargement.

### Histologic analysis

Histologic analysis was performed with the help of Masson–Goldner and van Kossa coloring. The embedded slices had to be prepared with several washing steps with xylol, 2-methoxyethlyacetat, acetone and aqua dest. to separate polymerate and bone preparation and to rehydrate samples. Masson–Goldner staining is a trichrome staining with 3 dye molecules in different sizes (azophloxin, organge G, lightgreen SF) to picture muscle fiber, collagen fiber, bone, fibrin and erythrozytes selectively. Staining of the nucleus was performed with Weigert's iron hematoxylin staining. Van Kossa staining is based on silver nitrate, that changes calcium against silver ions, so that mineralized tissue can be differentiated by unmineralised tissue. With the help of several washing steps slices were silvered by 5% silver nitrate followed by dehydration. Masson–Goldner stained slices were digitized, blinded and scored by three independent observers with the help of a modified Petty et al. score after deviding each sample into 4 regions of interest (ROI: proximal metaphysis, proximal diaphysis, distal diaphysis, distal metaphysis)^35–37^. The Petty et al. score is used for analysis of in-vivo models and guarantees a good option to evaluate inflammation: observers evaluated the formation of abscesses, of sequestra, destruction or widening of the corticalis and the overall impression of the bone with a score system with a maximum of 24 points for each tibia. A high score point corresponds to a high degree of destruction. Van Kossa-stained samples were analysed and evaluated by image analysis system BX51, Olympus and by Image Pro Plus®-software in order to determine the percentage of mineralized bone.

### Radiologic analysis

Rats' tibiae of all groups (n = 120) were devided into three ROI: proximal metaphysis, diaphysis and distal metaphysis. All tested animals were included for the radiologic analysis (n = 120). X-rays were taken at the day of the nail exchange, at each examination day and at the day of sacrification. By using a modified An et al. score^37^ X-rays were evaluated in comparison to the first postoperative X-ray by three blinded observers. The observers evaluated the level of destruction of every ROI following fixed criteria and a score system that assigned 0 points for no destruction, 1 point for 1 osteolysis and 2 points three oder more osteolysis. Observers assigned 3 points for the formation of sequestra, spontaneous fractures, deformation of the diaphysis, soft tissue swelling and radiologic overall impression so that a maximum of 33 point could be reached for one tibia.

### Statistical analysis

Statistical analysis was performed by IBM SPSS Statistics program for statistical analysis [IBM SPSS Statistics for Windows, Version 15.0. Chicago, USA: IBM Corp] and R version 4.1.0 (2021–05-18, R Foundation for statistical computing, Vienna, Austria). Correlation between continuous variables were examined using Spearman-correlation analysis and differences between groups were tested using two-sided Mann–Whitney U test and the Kruskal–Wallis test. Differences between the groups for categorical variables were tested via two-sided Chi-square test. To evaluate the inter-observer reliability of the three observes, a Fleiss Kappa was determined. All statistical analysis were purely explorative and an adjustment for multiple testing problem was not performed. P-values p < 0.05 were considered as statistically noticeable.

## Results

### Physical examination

**Figure 1:**
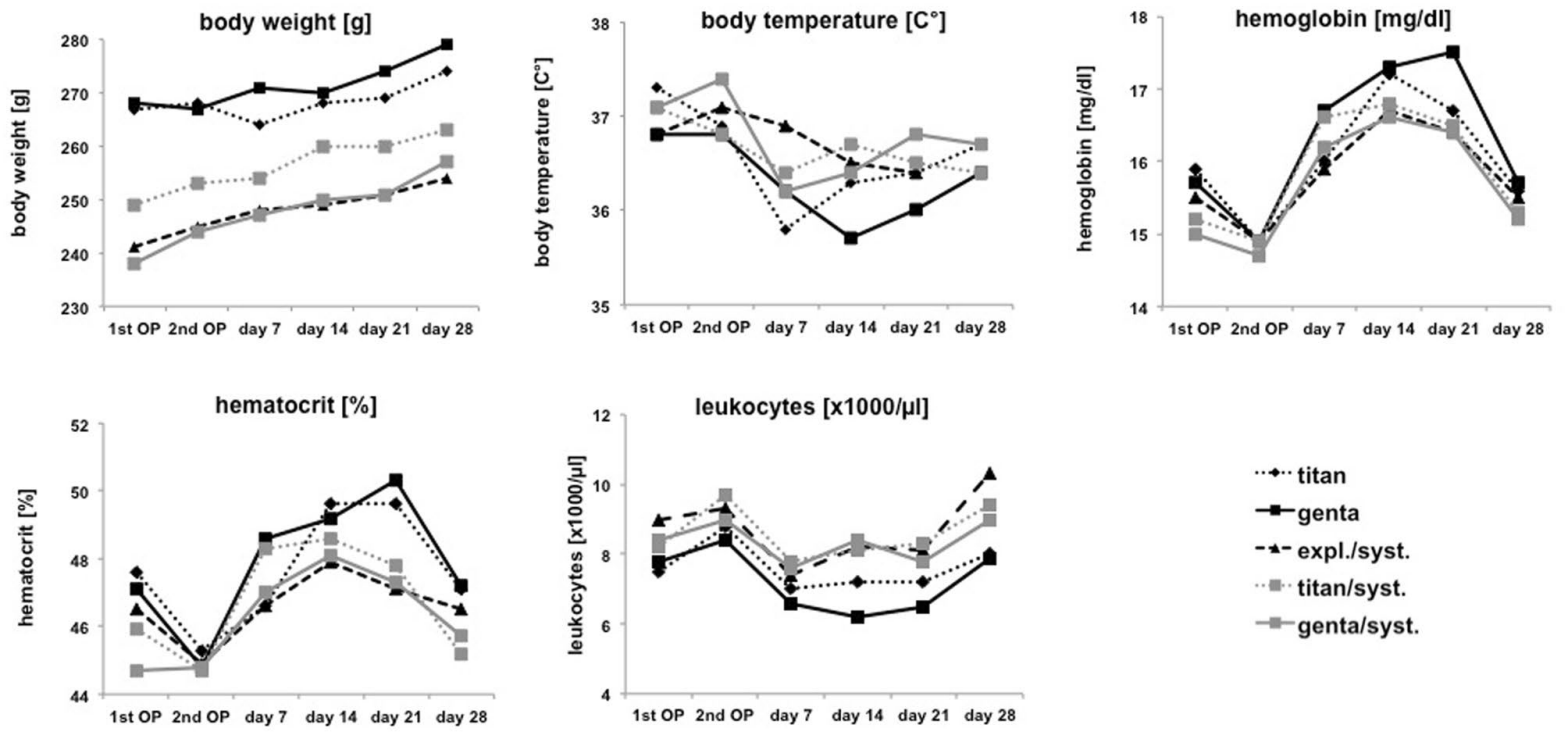
The results of the physical examination at the timepoint of first and second operation followed by physical examination at days 7, 14, 21 and 28 after the second operation and of bloodanalysis on those days showed stable results during the course of examination concerning the parameters body weight [g], body temperature [C°], hemoglobin [mg/dl], hematocrit [%] and leukocytes [× 1000/µl].

The physical examination at the timepoint of first and second operation followed by physical examination at day 7, 14, 21 and 28 showed a stable and continuous gain of body weight of each animal during the time course (238–279 g). None of the animals showed a loss of weight ≥ 10% (Fig. 1). The average body temperature (Fig. 1) demonstrated a physiological range (35.8–37.3 g) without any decrease as a possible sign of septicemia. The bloodanalysis (Fig. 1) with special focus on hemoglobin [mg/dl], hematocrit [%| and leukocytes [× 1000/µl] demonstrated an intial postoperativ decrease of hemoglobin (14.7–14.9 mg/dl) in all groups followed by an increase from day 7 to day 28 (15.9–16.7 mg/dl) with variations in a physiological range. According to hemoglobin the hematocrit also showed an initial decrease (44.7–45.3%) in all groups followed by an increase from day 7 to day 28 (46.6–50.3%). The course of leukocytes showed an increase at the beginning in all study groups followed by a decrease and an increasing tendency again at day 28 with a physiological range from day 1 to day 28 (7.5–10.3 × 1000/µl). Supplement 1 shows means and standard deviations in addition to Fig.1.

### Microbiologic analysis

**Figure 2:**
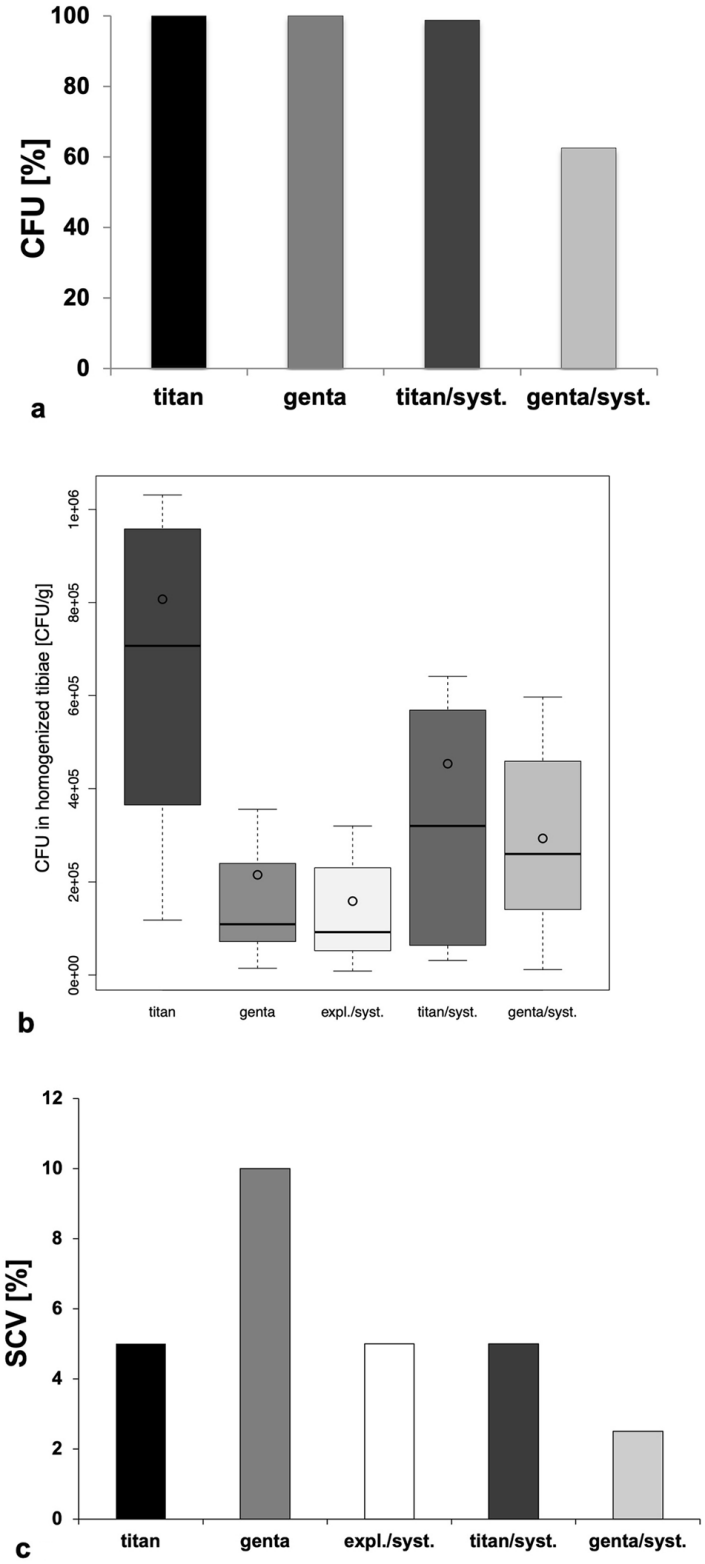
(**a**) Plated k-wires at day 28 show an infection rate of 100% in group titan and genta. At day 28 examination group titan/syst. resulted in an infection rate of 98.75%. Group genta/syst. resulted with the lowest infection rate with a percentage of 62.5%, which is demonstrated in Fig. 1a. (**b**) The evaluation of the homogenized tibiae showed a high amount of CFU in the examination group that was only treated with a titanium implant. The amount of CFU was noticeably higher than in the groups genta, expl./syst. and genta/syst. followed by group titan/syst. without significant difference. A low amount of CFU was counted in the groups genta, expl./syst. and genta/syst. (**c**) Evaluation of homogenized bone occurred with a special focus on the development of SCVs. Results show synthesis of SCVs in 4 animals of group genta, 2 animals of group titan, expl./syst. and titan/syst. and 1 animal of group genta/syst.

The number of colony forming units (CFU) is determined out of the inoculum, the smear tests, the plated implants as well as the incubated implants, the homogenized tibiae (n = 40) and kidneys (n = 80) and the heartblood (n = 40). All plated implants (n = 120), which were plated at the day of the nailexchange, showed an infection rate of 100% in all examination groups. Plated k-wires at day 28 (n = 96) showed an infection rate of 100% in group titan and genta. At day 28 examination group titan/syst. resulted with an infection rate of 98.75%. Group genta/syst. showed the lowest infection rate with a percentage of 62.5% (Fig. 2a). In these cases, the microbiologic analysis of the smear test and of the homogenized tibia also resulted with no bacterial growth. All analysed implants with bacterial growth showed cloudiness of glucose bouillon after incubation. Formation of gas, discoloration and sediment could not be proven in any of the samples. The homogenized tibiae of the examination group that was only treated with a titanium implant showed a high amount of CFU (Fig. 2b), that is noticeable higher than in the groups genta (p = 0.012), expl./syst. (p = 0.005) and genta/syst. (p = 0.011) followed by group titan/syst. without noticeable difference. A low amount of CFUs was counted in the groups genta, expl./syst. and genta/syst. Evaluation of homogenized bone occurred with a special focus on the development of SCVs (Fig. 2c). Results showed synthesis of SCVs in 4 animals of group genta, 2 animals of group titan, expl./syst. and titan/syst. and 1 animal of group genta/syst. Examination of homogenized kidneys and heartblood offered 1 infected bloodculture in group titan and genta and 1 infected kidney in group genta. In total, 7.5% of the infected animals showed a generalized infection.

### Immunohistochemical analysis

**Figure 3:**
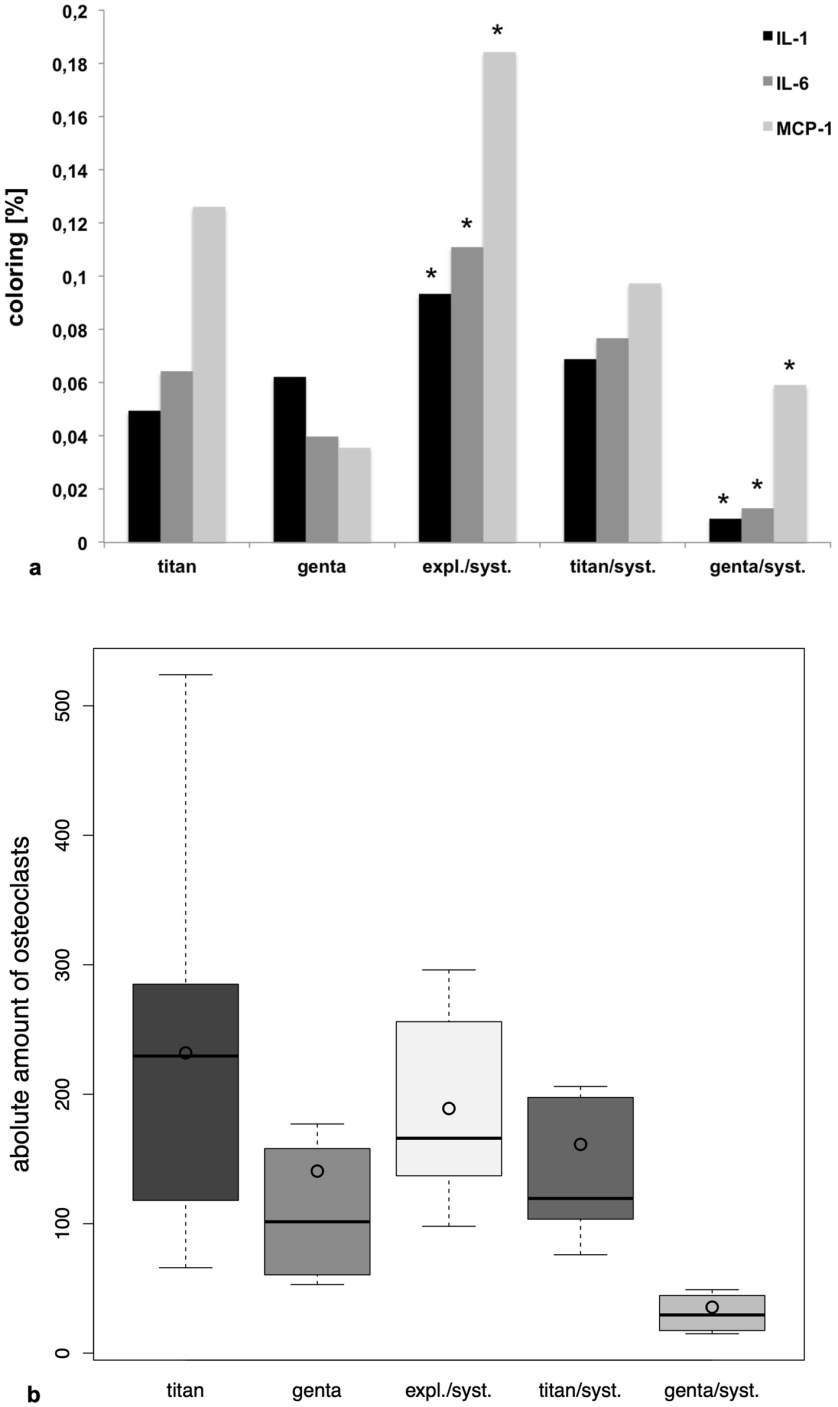
(**a**) Quantitativ analysis of group genta/syst. resulted with the significant lowest coloring concerning IL-1, IL-6 and MCP-1. Expl./syst. showed most signs of infection with a noticeable difference to the other examination groups. Furthermore expl./syst. reached the highest score after descriptiv analysis, which was lower than the score points of other study groups. On the other hand, group genta/syst. resulted with the lowest score compared to the other examination groups. (**b**) Results of the cytochemical analysis after TRAP-coloring. Study group genta/syst. resulted with the lowest number of osteoclasts and with a noticeable difference to the other groups. The highest amount of osteoclasts could be detected in examination groups titan and expl./syst.

Quantitativ analysis of group genta/syst. resulted with the noticeable lowest coloring concerning IL-1, IL-6 and MCP-1 (Fig. 3a). Expl./syst. showed most signs of infection with a noticeable difference to the other examination groups. Furthermore, expl./syst. reached the highest score after descriptive analysis (IL-1: 10.5; IL-6: 10.33; MCP-1: 10.67), which was higher than the score points of other study groups. Group genta/syst. resulted with the noticeable lowest score (IL-1: 2; IL-6: 1.67; MCP-1: 3.83) compared to the other examination groups. In addition Supplement 2 shows a representative picture of immunohistochemical coloring of each group concerning IL-1, IL-6 and MCP-1.

### Cytochemical analysis

After TRAP-coloring study group genta/syst. resulted in the lowest number of osteoclasts and with a noticeable difference to the other groups (p = 0.001–0.003). The highest amount of osteoclasts could be detected in examination groups titan and expl./syst. (Fig. 3b).

### Histologic analysis

**Figure 4:**
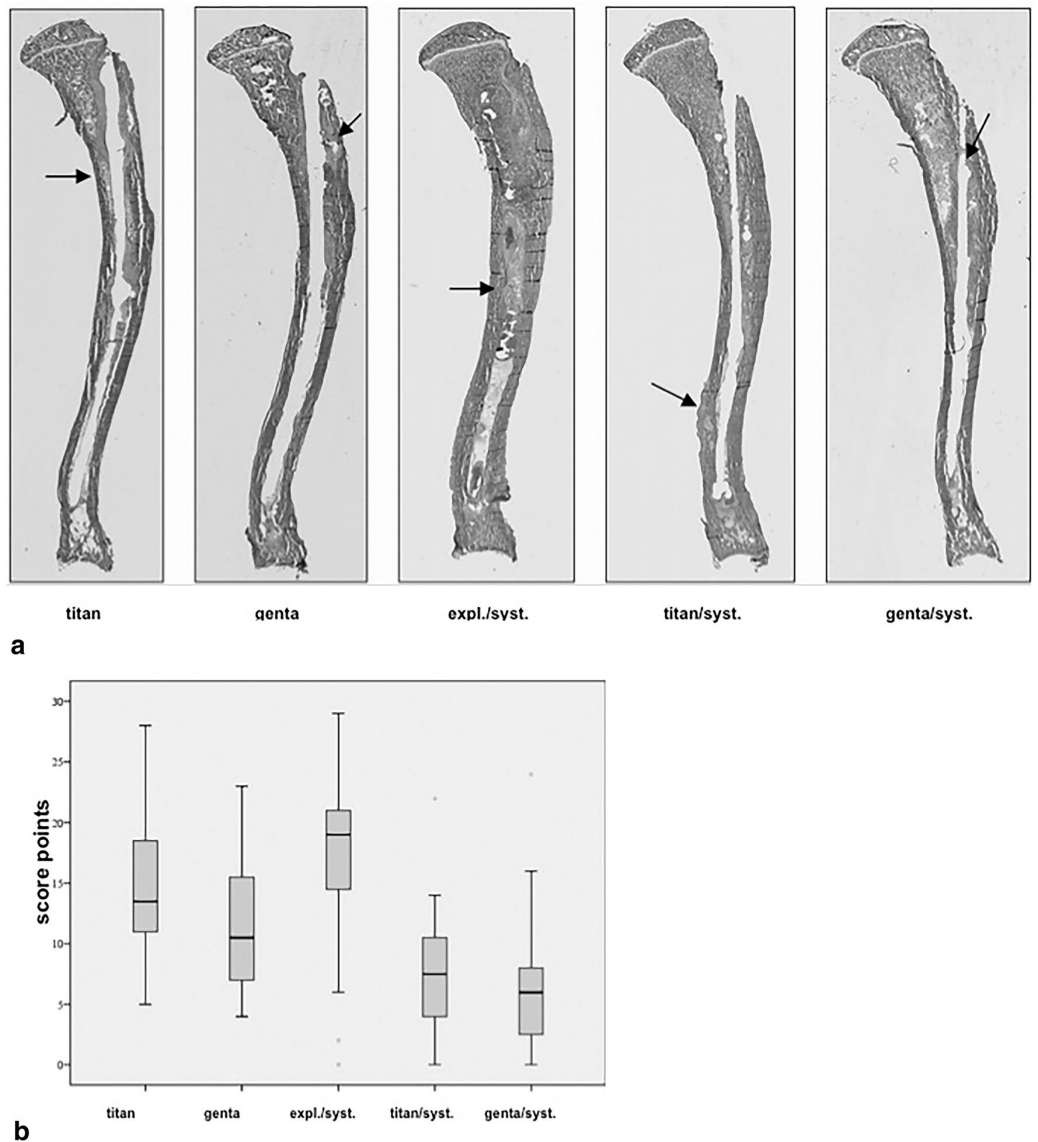
(**a**) Shows representative slices of a tibia of each group colored with Masson–Goldner for histological analysis. Group expl./syst. shows massive signs of bone infection like osteolyses, abscesses and formation of sequestra (arrow). Group titan reveals increase of granulocytes and osteolysis as well genta and titan/syst. and also shows periosteal enlargement. Only group genta/syst. shows few signs of infection with little formation of osteolysis and a non-infected drill channel. (**b**) Statistical analysis of the development of histological signs of infection of all data demonstrated as boxplots.

**Figure 5:**
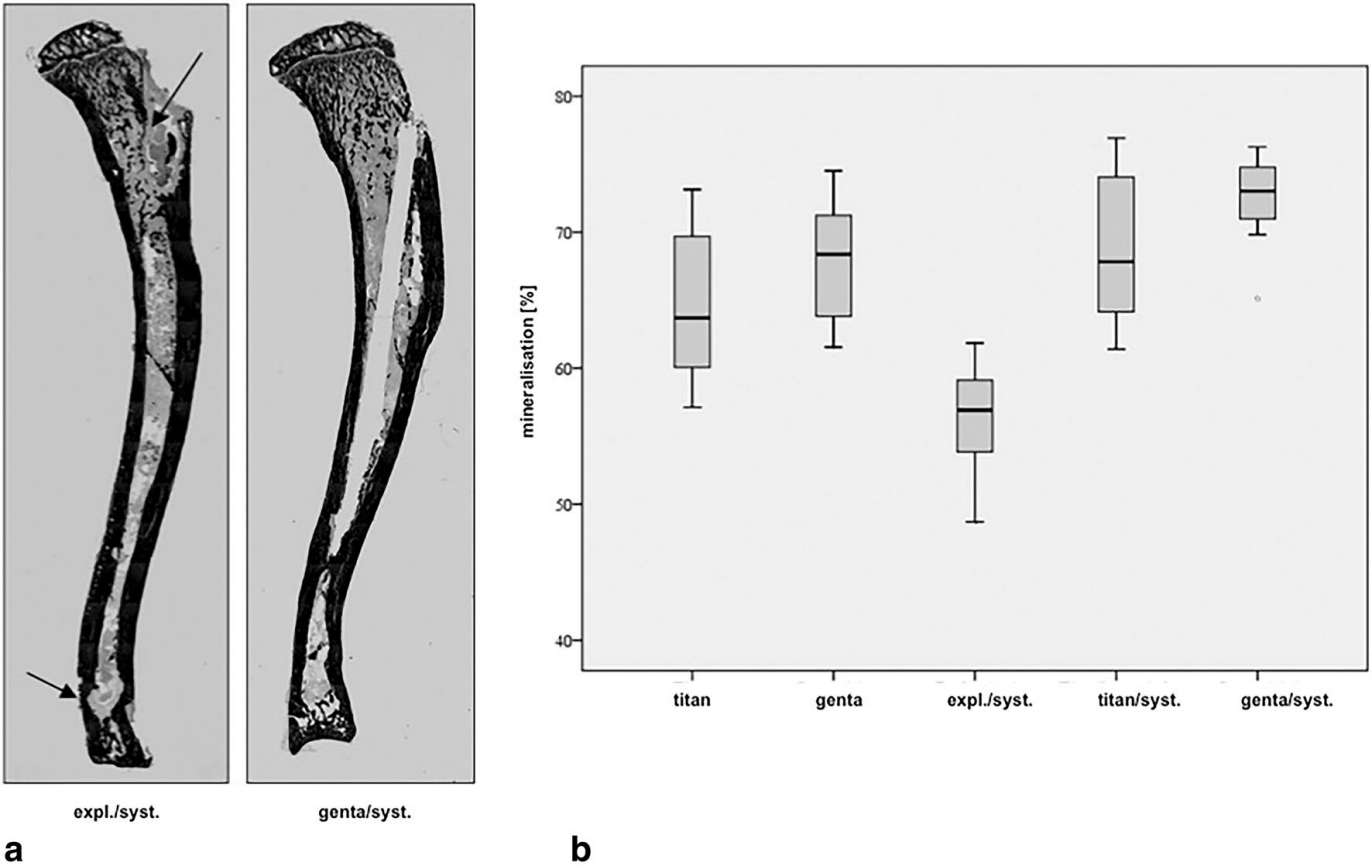
(**a**) Shows representative slices of a tibia of the groups expl./syst. and genta/syst. colored with van Kossa coloring. Group genta/syst. shows a higher grade of bone mineralisation. (**b**) Statistical analysis of the grade of mineralization of bone of each examination group demonstrated as boxplots.

Histologic analysis was performed with Masson–Goldner and van Kossa coloring. Masson–Goldner colored samples showed different stages of implant-associated infection concerning study treatment. A representative example of Masson–Goldner coloring is shown in Fig. 4a. The evaluation by a modified Petty et al.^34–36^ score ranked the group expl./syst. with the highest score points and noticeable more signs of osteomyelitis than genta (p = 0.001), titan/syst. and genta/syst. (p < 0.001). Group titian resulted in noticeable higher scores compared to titan/syst. and genta/syst. (p < 0.001) as well as genta compared to titan/syst. (p = 0.008) and genta/syst. (p < 0.001). Furthermore, genta was scored with noticeable lower infection signs than expl./syst. (p = 0.001). Examination group titan/syst. resulted with noticeable less infection than genta (p = 0.008), titan and expl./syst. (p < 0.001). Overall genta/syst. was scored with lowest signs of infections compared to all others study groups (p < 0.001). Figure 4b demonstrates histological results graphically. The evaluation of the inter-observer-reliability of 3 observers resulted in accordance to p = 0.01. Van Kossa-stained samples were analysed and evaluated by image analysis system BX51, Olympus and by Image Pro Plus®-software in order to determine the percentage of mineralized bone (Fig. 5a,b). Group expl./syst. was scored with a mineralisation of 56.93% and ranked noticeable lower compared to titan (p = 0.007), genta, titan/syst. and genta/syst. (p < 0.001). Group Titan, with a grade of 63.7%, resulted noticeable lower than genta/syst. (p = 0.015) and higher than expl./syst. (p = 0.007). Groups titan/syst. (67.85%) and genta (68.37%) showed noticeable higher mineralization than expl./syst. (p < 0.001). Examination group genta/syst. was evaluated with the highest grade of mineralization (73.02%) compared to titan (p = 0.015) and expl./syst. (p < 0.001).

### Radiologic analysis

**Figure 6:**
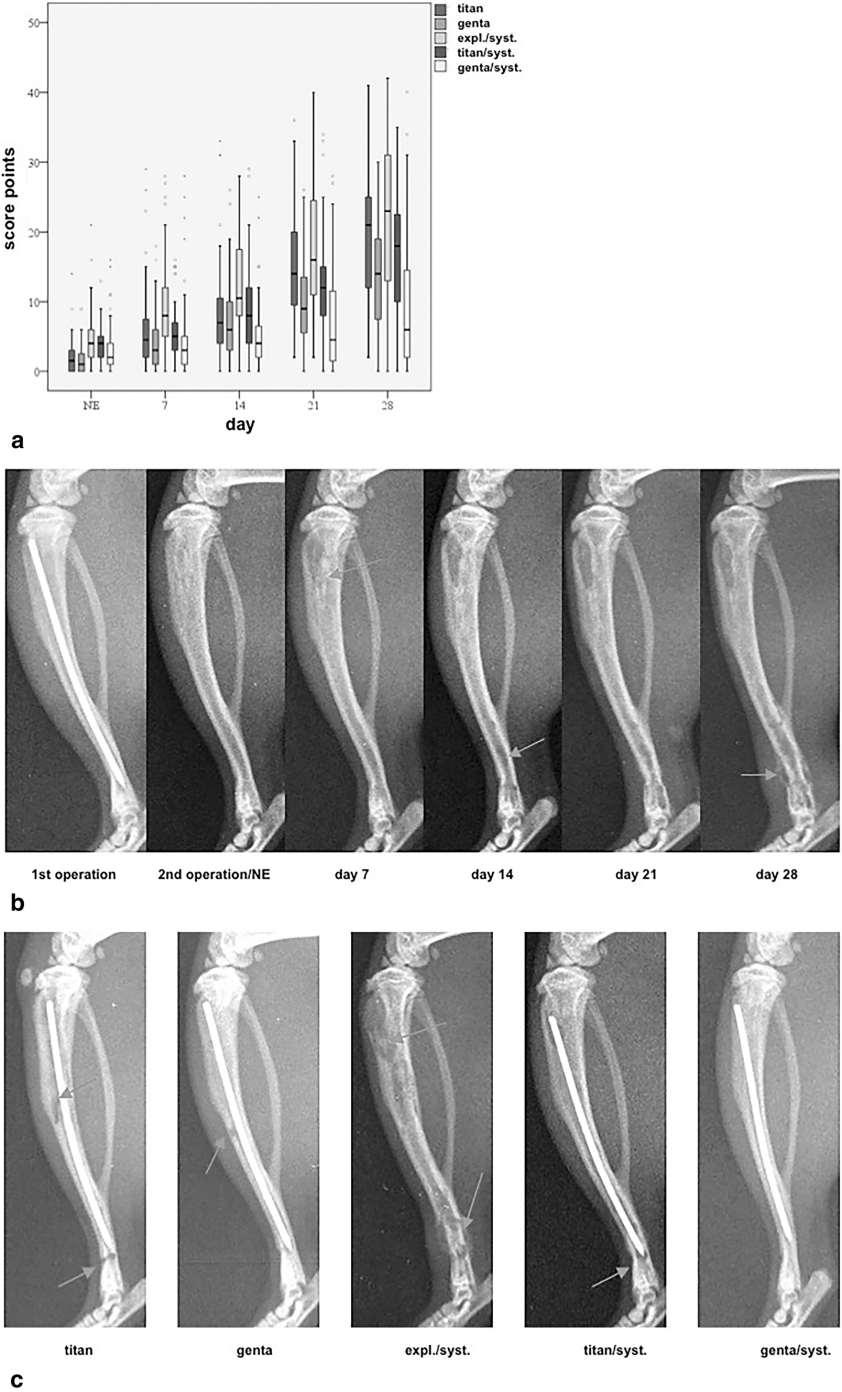
(**a**) Statistical analysis of the development of radiological signs of infection of all data demonstrated a significant change in all groups during the course of study till day 28 and also in between the study groups (p < 0.001). Here, the scorepoints of each examination group at each examination day after evaluation with the An et al. score are demonstrated. (**b**) Shows representative lateral x-rays of the left tibia of the group expl./syst. and gives an impression of the development of osteolysis during study course. The study group expl./syst. was scored with the highest points. First signs of osteolysis can be detected at day 7 (arrow). At day 14, signs of infection can be observed at the distal part of the tibia (arrow). The whole structure of the tibia has changed at day 28 due to bone infection with osteolysis of the complete bone, periosteal infection and pathologic fracture. (**c**) Shows representative lateral x-rays of the left tibia of each study group at the endpoint of the study at day 28. Signs of bone infection can be detected in all examination groups at day 28, especially group expl./syst. reveales massive osteolysis. Just genta/syst. shows very low signs of bone infection.

The first osteolysis could be observed at day 7 in all groups. Clear signs of osteolysis could be found in group expl./syst. Group expl./syst. showed first periosteal reaction at day 7. Group titan and genta resulted in the first periosteal reaction at day 21, group titan/syst. and genta/syst. at day 28. Soft tissue swelling occurred in all groups with high development in groups titan and expl./syst. First bone deformations could be evaluated in group expl./syst. at day 14 and at day 21 in groups titan and genta. Group expl./syst. also revealed pathological fractures at a later study time point as well as sequestra. Statistical analysis of radiological signs of infection demonstrated a noticeable change in all groups during the course of study until day 28 and also inbetween the study groups (p < 0.001) (Fig. 6a). At the day of nail exchange, all groups are scored on a low level. At day 7 group expl./syst. was scored noticeable higher (p < 0.001) compared to the other study groups. At day 28 group expl./syst. is evaluated with the highest score points with a noticeable difference (genta p < 0.001, genta/syst. p < 0.001, titan/syst. p = 0.002) and showed clear signs of infection. Group genta/syst. showed the lowest score points, which are noticeably lower compared to all other study groups (p < 0.001). Figure 6b gives an impression of the development of osteolyses during the study course in the study group expl./syst. with the highest scorepoints. Figure 6c shows representative lateral x-rays of the left tibia of each study group at the endpoint of the study at day 28 with massive osteolysis, especially in group expl./syst. The evaluation of the inter-observer reliability of 3 observers results in accordance (p = 0.01).

## Discussion

In the field of osteomyelitis, it is difficult to manage a valid clinical study because of various influencing factors. Therefore, in-vivo models with a definite simulation of the pathophysiology of diseases are a good option as those are comparable, reproducible, and controlled^38^. In 1884 Rodet established the first osteomyelitis model in-vivo^39^. Implant-associated infections and their course with serious consequences necessitate further investigations to evaluate treatment options and to match experimental and clinical workflow. Analogous to clinical routine, our study is conducted concerning standard hygiene measures that have to be followed during every surgical intervention. As mentioned above, surgical debridement is known to be the most effective treatment component and leads to improved wound healing^3,11^, so that intensive debridement is performed with 5% Lavasept®-solution in the study. Nevertheless, actual literature discusses that the efficacy of surgery alone in cases of implant-associated infections is not sufficient. A combined therapy out of surgery and antibiotic treatment is necessary^3,11^. Klemm and Wildfeuer could show in their studies that local antibiotic carriers like bone cement and PMMA-chains decrease the incidence of wound infections, especially in combination with a systemic perioperative antibiotic prophylaxis^22,24^. Furthermore, Lucke et al. demonstrated the effectiveness of gentamicine-coated implants to prevent the development of osteomyelitis in an in-vivo model^35^. In order to prevent the formation of resistances, PMMA-chains or cement-spacers have to be removed after a short period of time. The advantage of the coating substance PDLLA, which also serves as the antibiotic carrier in this study, is that it is completely removed hydrolytically, which leads to a complete release of the active substance in a defined time limit^27^. On the basis of the established in-vivo infection model by Fuchs et al.^31^ the study analyses the influence of a post-infectious implant exchange one week after infection and the infection progress afterwards in combination with a systemic versus a local antibiotic treatment in-vivo. Even though the two-stage material exchange is the gold standard in clinical routine in cases of implant-associated infections^40^, this study evaluates the one-period exchange with consideration of operation risks, treatment costs, and treatment duration. During the examination, microbiological analysis ensured a manifest infection after one week as every removed and plated implant showed bacterial growth. Postoperatively every examination group showed an initial decrease of hemoglobin [g/dl] and hematocrit [%] due to the operation, followed by an increase and a regeneration at day 28. Immunohistochemical analysis is not yet spread and further studies are missing. Our investigation shows that it is a good tool to correlate collected date and evaluate the intensity of infection. With the help of randomization and three independent observer results became valid. X-ray is known to be the standard diagnostic tool in osteomyelitis as a base line to monitore infection in addition to MRI^41,42^. First radiological conspicuousness could be detected 10 to 14 days after clinical symptoms occur^43^, which coincides with the study results that were analysed by a modified An et al. score^37^. Histological analysis was performed by a modified Petty et al. score^36^, that is used for analysis of in-vivo models and guarantees a good option to evaluate inflammation^34–36,44^. Radiological results show that infection progression could not be stopped in any examination group, but differed in its severity concerning the examination group. At day 28 the test group without any implant and just systemical antibiotic treatment showed most signs of infection in radiological as well in histological anaylsis, which leads to the conclusion, that the implant removal in combination with a systemic antibiotic treatment is not an efficient option as bacteria might survive and colonise the open drill channel plus mechanical stability is missing, which is one of the most important aspects in infection treatment. Radiological and histological results also demonstrate a significant increase of infection in the examination group, in which an uncoated implant is re-implanted without any systemic treatment as the new uncoated material is a new surface, that can be covered by bacteria and which leads to excacerbation of infection^17^. Examination groups with either a local antibiotic coating or without coating but with a systemic treatment show similar results and less development of infection compared to non-antibiotic treatment locally or systemically. Results show that the combination of local gentamicin coating and systemic antibiotic therapy is most efficient and prevents dramatic infection progress radiologically and histologically. Findings can be supported by analysis of bone mineralization, that reveals higher rates of mineralization in less infected bone as *S. aureus* induces TNF-α, IL-1 and IL-6 synthesis, which is the reason for disturbed bone remodeling: osteoclast activity is increased as well as bone resorption, whereas osteoblast activity is decreased combined with decreased mineralization and matrix production. Studies concerning the role of cytokines might be important for further treatment options^2,45^. Overall, infection progression could not be inhibited by any kind of treatment. In conclusion, local and systemic treatments are known to be an efficient approach, whereas the therapy was not able to cure infection in this model. 11 animals showed SCVs as it is known for gentamicine to promote SCV-synthesis^46^. Therefore, rifampicin might be potent alternative in combined therapy as it is practiced in clinical daily routine^47^. The investigation shows a clear advantage of the combined local and systemic antibiotic treatment in contrast to the implant removal and to a non-combined antiobtic therapy. These findings can be supported by literature. Yokogawa and Stavrakis also emphasize the role of implant exchange^48^ and implant coating with antibiotic substances^49^. In accordance to our results, Masters et al. reported, that the removal of infected implants is essential. Furthermore, they emphazise the importance of implant coating and the fact, that each case of osteomyelitis is unique and that each therapy has to be planned individually. The role of one-stage vs. two-stage revision remains unclear and needs further investigation^6^.

Limitations of our study are that we focused clearly on the influence of implant exchange and implant coating. Therefore, we did not include a control group without any operation and without any implant. We just used one antibiotic substance for local and systemic treatment, which has to be modified for further investigation. Due to an in-vivo model we have to deal with a relatively small group size. Futhermore, the one-period exchange has to be evaluated in contrast to two-stage implant exchange (Supplementary Figs. 1 and 2).

Overall, our established in-vivo rat-model is valid and reproducible and therefore provides the opportunity to investigate further treatment options, potent treatment alternatives and influencing factors concerning the therapy of implant-associated infections with the focus on the combination of local and systemic antibiotic treatment with less severe infection progress as our results show. In consideration of the limits of our study, further in-vivo investigation is necessary.

## Supplementary Information

Supplementary Figure 1.

Supplementary Figure 2.
