# Supplementary material for: Establishment of a reliable in-vivo model of implant-associated infection to investigate innovative treatment options
Source: Sci Rep. 2022 Mar 10;12:3979. doi: 10.1038/s41598-022-07673-8 (PMC8913616; doi:10.1038/s41598-022-07673-8)
Supplement: Supplementary file 1 — Supplementary Figure 1. [file 41598_2022_7673_MOESM1_ESM.pdf]

Establishment of a reliable in-vivo model of implant associated infection to investigate innovative treatment options

Kreis C[1]*, Aschenbrenner FK[2], Günther D[3], Tholema-Hans N[1], Koeppe J[4], Rosslenbroich SB[1], Raschke MJ[1], Fuchs T[1, 5]

[1] Department of Trauma, Hand and Reconstructive Surgery, University Hospital of Muenster, Muenster, Germany

[2] Department of Anesthesia, Hospital Lippe Detmold, Germany

[3] Department of Orthopaedic Surgery, Trauma Surgery and Sports Medicine, Cologne Merheim Medical Center, Witten/Herdecke University, Cologne, Germany

[4] Institute of Biostatistics and Clinical Research, University of Muenster, Germany

[5] Department of Trauma and Reconstructive Surgery, Vivantes Clinic Friedrichshain, Berlin, Germany

	1st OP		2nd OP		day 7		day 14		day 21		day 28	
**body weight [g]**	MW	SD	MW	SD	MW	SD	MW	SD	MW	SD	MW	SD
**titan**	267	21,9	268	21,2	264	20	268	22,3	269	18,7	274	20
**genta**	268	24,2	267	21,5	271	22,4	270	22,4	274	21,9	279	21,8
**expl./syst.**	241	9,9	245	11,1	248	11,9	249	10,7	251	9,8	254	10,8
**titan/syst.**	249	18,8	253	20,1	254	19,8	260	19,5	260	18,1	263	19,6
**genta/syst.**	238	9,5	244	13,2	247	11	250	10,3	251	12,1	257	10,5
**body temperature [C°]**												
**titan**	37,3	0,6	36,9	0,7	35,8	1,2	36,3	0,6	36,4	0,6	36,7	0,6
**genta**	36,8	0,6	36,8	0,5	36,2	0,7	35,7	1	36	0,6	36,4	0,7
**expl./syst.**	36,8	0,9	37,1	0,8	36,9	0,8	36,5	0,7	36,4	0,6	36,6	0,9
**titan/syst.**	37,1	1	36,8	0,8	36,4	0,8	36,7	0,6	36,5	0,6	36,4	0,6
**genta/syst.**	37,1	0,8	37,4	0,6	36,2	0,6	36,4	0,8	36,8	0,6	36,7	0,6
**hemoglobin [mg/dl]**												
**titan**	15,9	0,6	14,9	0,6	16	2,8	17,2	0,7	16,7	2,7	15,6	0,5
**genta**	15,7	0,7	14,9	0,7	16,7	0,6	17,3	0,6	17,5	0,8	15,7	0,8
**expl./syst.**	15,5	0,8	14,9	0,7	15,9	1,1	16,7	0,9	16,4	0,8	15,5	0,8
**titan/syst.**	15,2	1,2	14,9	0,6	16,6	1,1	16,8	1,1	16,5	0,9	15,3	0,6
**genta/syst.**	15	0,8	14,7	0,7	16,2	0,7	16,6	0,6	16,4	0,6	15,2	0,5
**hematocrit [%]**												
**titan**	47,6	1,9	45,3	1,8	46,6	7,4	49,6	2,4	49,6	1,3	47,1	2
**genta**	47,1	1,8	44,8	1,6	48,6	1,7	49,2	2,3	50,3	1,8	47,2	1,8
**expl./syst.**	46,5	2,3	44,9	2,3	46,6	2,6	47,9	2,2	47,1	1,9	46,5	2,1
**titan/syst.**	45,9	2,4	44,7	1,8	48,3	3,2	48,6	2,9	47,8	2,5	45,2	1,7
**genta/syst.**	44,7	2,4	44,8	2,3	47	2	48,1	2,1	47,3	1,9	45,7	1,8

**leukocytes [x1000/µl]**												
**titan**	7,5	2	8,8	2,7	7	2,3	7,2	2	7,2	1,9	8	1,9
**genta**	7,8	1,6	8,4	1,8	6,6	1,2	6,2	1,7	6,5	1,4	7,9	1,4
**expl./syst.**	9	1,8	9,3	1,3	7,4	1,1	8,2	1,6	8,1	1,5	10,3	1,8
**titan/syst.**	8,2	1,2	9,7	1,9	7,8	1,8	8,1	1,9	8,3	2,8	9,4	1,6
**genta/syst.**	8,4	1,7	9	2	7,6	1,6	8,4	1,5	7,8	1,5	9	1,8

Supplement Figure 1: Means and standard deviations in addition to Figure 1.
