# Supplementary material for: Establishment of a reliable in-vivo model of implant-associated infection to investigate innovative treatment options
Source: Sci Rep. 2022 Mar 10;12:3979. doi: 10.1038/s41598-022-07673-8 (PMC8913616; doi:10.1038/s41598-022-07673-8)
Supplement: Supplementary file 2 — Supplementary Figure 2. [file 41598_2022_7673_MOESM2_ESM.pdf]

Establishment of a reliable in-vivo model of implant-associated infection to investigate innovative treatment options

Kreis C[1]*, Aschenbrenner FK[2], Günther D[3], Tholema-Hans N[1], Koeppe J[4], Rosslenbroich SB[1], Raschke MJ[1], Fuchs T[1, 5]

[1] Department of Trauma, Hand and Reconstructive Surgery, University Hospital of Muenster, Muenster, Germany

[2] Department of Anesthesia, Hospital Lippe Detmold, Germany

[3] Department of Orthopaedic Surgery, Trauma Surgery and Sports Medicine, Cologne Merheim Medical Center, Witten/Herdecke University, Cologne, Germany

[4] Institute of Biostatistics and Clinical Research, University of Muenster, Germany

[5] Department of Trauma and Reconstructive Surgery, Vivantes Clinic Friedrichshain, Berlin, Germany

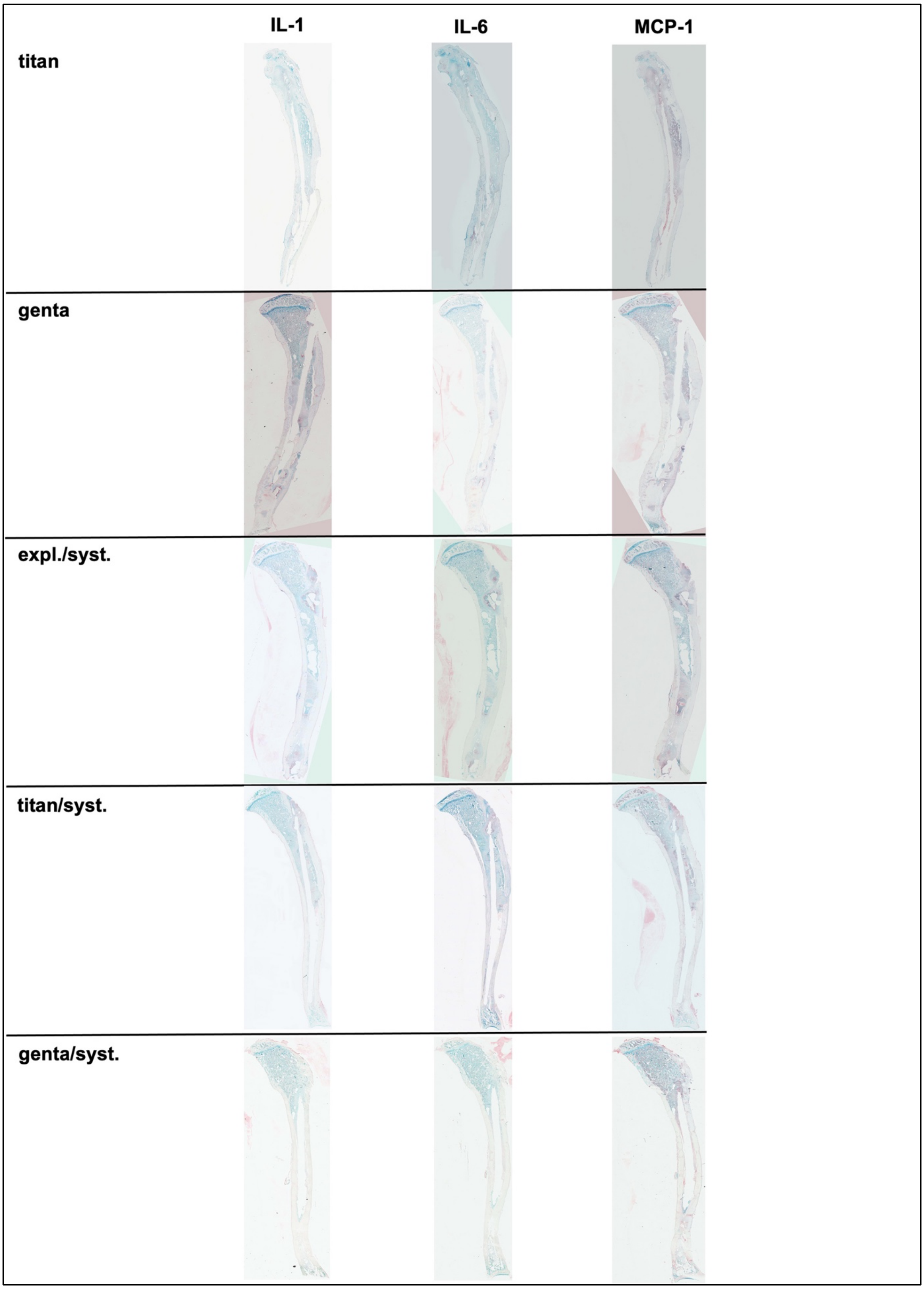

Supplement Figure 2: Representative pictures of immunohistochemical coloring of each group concerning IL-1, IL-6 and MCP-1
